# Inhibition of hippocampal cyclin‐dependent kinase 5 activity ameliorates learning and memory dysfunction in a mouse model of bronchopulmonary dysplasia

**DOI:** 10.1111/cns.14185

**Published:** 2023-03-25

**Authors:** Fang‐Fei Tao, Zi‐Yu Wang, Ying Wang, Qian‐Ru Lv, Peng‐Peng Cai, Hai‐Wen Min, Jian‐Wei Ge, Chun‐Yu Yin, Rui Cheng

**Affiliations:** ^1^ Department of Neonatal Medical Center Children's Hospital of Nanjing Medical University Nanjing China; ^2^ Nanjing Medical University Nanjing China; ^3^ Department of Neurology, Drum Tower Hospital, Medical School and The State Key Laboratory of Pharmaceutical Biotechnology Nanjing University Nanjing China

**Keywords:** bronchopulmonary dysplasia, CDK5, hippocampus, learning and memory, synaptic plasticity

## Abstract

**Aims:**

Oxygen therapy plays a vital role in the development of bronchopulmonary dysplasia (BPD), which is the independent risk factor for neurodevelopment deficits in premature infants. However, the effect of hippocampal cyclin‐dependent kinase 5 (CDK5) on BPD‐associated neurodevelopment deficits is not fully understood.

**Methods:**

Mice were placed in a hyperoxia chamber from postnatal Day 1 to Day 7. Hematoxylin and eosin staining was used to evaluate the lung histomorphological characteristics. Learning and memory functions of mice were detected by Morris water maze. TUNEL staining was applied to measure the number of apoptotic cells. The expression of CDK5, apoptosis‐related protein, and neuroplasticity‐related proteins were analyzed by Western blot. Golgi staining was used to assess the structure of dendritic spines.

**Results:**

Hyperoxia‐induced BPD mice showed a long‐term learning and memory dysfunction, more severe neuronal apoptosis, and a decline of synaptic plasticity. Inhibition of CDK5 overactivation ameliorated cognitive deficits, neuronal apoptosis, and synaptic plasticity disorders in BPD mice.

**Conclusions:**

This study first found a vital role of CDK5 in BPD‐associated neurodevelopmental disorders. Inhibition of CDK5 overexpression could effectively improve cognitive dysfunctions in BPD mice, which indicated that hippocampal CDK5 may be a new target for prevention and treatment in learning and memory dysfunction of BPD.

## Graphical abstract

This study first found that hyperoxia induced a dramatic increase hippocampal CDK5 expression in both short‐term and long‐term effects in BPD mice. Hyperoxia‐induced BPD mice showed a long‐term learning and memory dysfunction, more severe neuronal apoptosis, and a decline of synaptic plasticity. Inhibition of CDK5 overactivation ameliorated cognitive deficits, attenuated neuronal apoptosis, and synaptic plasticity disorders in BPD mice.

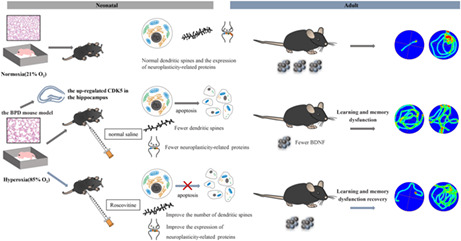

## 1 INTRODUCTION

Bronchopulmonary dysplasia (BPD) is a common disorder in premature infants characterized by simplified alveolar development and pulmonary microvascular injury. With the development of assisted reproductive technology and the progress of perinatal care, the delivery and survival rate of premature infants are significantly increased, with the increasing incidence of BPD.^1^ In recent years, a large number of clinical evidence have shown that BPD, as an independent risk factor for neurodevelopment in premature infants, not only causes brain injury in premature infants, but also increases neurodevelopmental disorders in preterm infants, including cognitive impairment.^2^, ^3^, ^4^Although basic studies have found that BPD rats have learning and memory dysfunction,^5^ the cellular and molecular mechanisms of BPD‐associated cognitive deficits remain to be further clarified.

Hippocampus is widely known for its crucial roles in spatial navigation, memory, and cognition.^6^ A new study shows that neurogenesis in the human hippocampus occurs mainly at 16–18 gestational weeks (GW), followed by axonogenesis at GW20–22 and function development at GW25–27.^7^ The hippocampal dentate gyrus (DG) possesses a strong neurogenesis capacity,^8^ which is neurogenic and susceptible to modulation by various environmental factors and neurotrophic factors, including brain‐derived neurotrophic factor (BDNF).^9^ As an abundant and well‐studied neurotrophic factor in the central nervous system, BDNF plays a critical role to adjust the structural and functions of synapses.^10^ In addition, hyperoxia exposure induced neuronal apoptosis in BPD rat model.^5^ However, currently, it is not clear whether the dysfunction of synaptic plasticity and neuronal apoptosis in the hippocampus of BPD.

Cyclin‐dependent kinase 5 (CDK5), a brain neuronal protein kinase that is highly expressed during embryonic development,^11^ has been extensively studied over the past 30 years.^12^ As a distinctive member of the CDK family,^13^ CDK5 is mainly involved in neurogenesis and apoptosis,^14^, ^15^axonal guidance,^16^ and synaptic plasticity,^17^ and also plays a regulatory role in neuropathic pain,^18^ affective disorders,^19^ circadian clocks,^20^ and learning and memory functions.^21^ In addition, according to existing research evidence, CDK5 induces the release of heat shock protein 70 (HSP70) by mediating the degradation of Bcl‐2‐associated athanogene 3 (BAG3) protein, leading to synaptic dysfunction in the pathogenesis of Alzheimer's disease.^22^ Meanwhile, CDK5 mediates nuclear translocation Sirtuin 2 (SIRT2) by directly phosphorylating SIRT2 residues, thereby promoting the death of dopaminergic neurons in a mouse model of Parkinson's disease.^23^ Furthermore, genetically reducing CDK5 ameliorates corticostriatal learning deficits and hippocampus‐dependent memory decline in mutant huntingtin knockout mice.^24^ In previous experiments, inhibition of CDK5 with Roscovitine (a specific inhibitor of CDK5) not only restored sevoflurane‐induced cognitive deficits by promoting SIRT1‐mediated autophagy,^25^ but also alleviated diabetes‐related cognitive dysfunctions by downregulating abnormal expression of CDK5.^26^ However, there are currently few studies on CDK5 in neonatal‐related diseases.

In this study, we explored the role of CDK5 in BPD‐associated cognitive deficits and explored the possible downstream signaling of hippocampus CDK5 mediating BPD‐associated cognitive impairments, revealing that Roscovitine treatment can effectively improve BPD‐related learning and memory dysfunction, which might provide an alternative strategy for the treatment of cognitive deficits in BPD.

## 2 METHODS

### 2.1 Animals and treatments

#### 2.1.1 Animals

Adult male and female wild‐type C57BL/6J mice (6–8 weeks) were purchased from the Model Animal Research Center of Nanjing University (Nanjing, China). These mice were freely bred to obtain newborn mice. Subsequently, neonatal mice, both male and female, were used for the experiments.

#### 2.1.2 BPD model

The procedure of BPD was designed as previously reported.^27^ Neonatal mice were randomly divided into two groups on the first day after delivery. The newborn mice in the BPD model group were placed in a hyperoxia chamber (85% O_2_) from postnatal Day 1 to Day 7 (P1–P7), while the newborn mice in the control group were exposed to air (21% O_2_) for the same time. During the experiment, the temperature (24°C) and humidity (50%–60%) were kept constant. The nursing dams of the two groups were exchanged every 24 h to feed the pups, in turn, to avoid the toxicity of hyperoxia in the dams and eliminate the maternal differences between groups. Subsequently, BPD and control neonatal mice were marked and mixed under air (21% O_2_) to reduce the experimental error. On the P21 after birth, the pups were separated from the nursing dams and developed independently.

#### 2.1.3 Drugs injection

The mice in the BPD group were intraperitoneally injected on the P3 and P7. The BPD + ROS group was intraperitoneally injected CDK5 inhibitor Roscovitine (ROS), while the BPD + NS group was intraperitoneally injected with the same dose of blank solvent (5% DMSO plus 95% normal saline). And, the concentration of ROS (10 mg/kg) was referred to literature.^28^ Obviously, the subsequent processing was consistent with the first stage. During the treatment procedures, approximately 0–3 mice in each group failed to survive.

#### 2.1.4 Behavioral test–Morris water maze (MWM)

To assess short‐term effects, we randomly sacrificed 15–18 pups per group at P14 for experimental analysis. Subsequently, for long‐term impact assessment, the remaining mice were subjected to MWM test at P56. Then, under deep anesthesia, at P70, the stage of somatic maturity, all mice were decapitated and brains were rapidly removed.

### 2.2 Lung histology and morphometry

At P14, five to eight pups per group were randomly selected for anesthesia with intraperitoneal injection of sodium pentobarbital, followed by tracheotomy and intubation under deep anesthesia, and then sacrificed for analysis. The left lung was perfused with 4% paraformaldehyde (PFA) under 25 cmH_2_O pressure through a tracheal tube for more than 15 min. The left lung was subsequently removed and fixed with 4% PFA overnight, and then embedded in paraffin. Finally, the lung tissue was cut into slices with per thickness of 5 μm. Quantitative analysis of lung histomorphology was performed with hematoxylin and eosin (H&E) staining as previously described.^29^ Five non‐overlapping photomicrographs were taken by a blinded investigator using an Olympus BX51 (Japan) microscope at different sections ×200 and ×500 magnifications, respectively, under the same lighting conditions and optical settings. Images were then analyzed by research‐based digital image analysis software (ImageJ, JAVA) to determine the number of alveoli and the mean linear intercept (MLI). The analysis results of five slices of left lung of each mouse were averaged.

### 2.3 Magnetic resonance image of the brain

Magnetic resonance imaging (MRI) was used to study the structure of the hippocampus in mice. Firstly, in the Small Animal Imaging Laboratory at Jiangsu Animal Experimental Center of Medical & Pharmaceutical Research, mice were fixed on a MRI machine (Biospec 7T/20 USR, Bruker) after isoflurane‐induced anesthesia, and the animals maintained breathing spontaneous. Scanned images of hippocampal tissue were obtained by T2‐weighted non‐contrast MRI (slice thickness 0.7 mm), and then, the structural regions of hippocampus on each slice were identified and defined according to the Mouse Brain in Stereotaxic Coordinates (Paxinos G, 2001). Finally, the hippocampal cross‐sectional area of each slice was quantified by ImageJ software (National Institutes of Health, USA). The hippocampal volume was calculated by multiplying the hippocampal cross‐sectional area of each slice by the slice thickness. The total volume of hippocampus could be estimated according to the following formula: Total Hippocampal Volume = $\sum_{f=1}^{n}d*A_{f}$, where $A_{f}$ is the cross‐sectional area on the f th slice through the mouse hippocampus, n indicates the total number of the considered slices and d the slice thickness (0.7 mm).^30^

### 2.4 Morris water maze

Morris water maze (MWM) test was performed to evaluate the learning and memory functions of mice in adulthood (P56‐P61).^31^ The pool with a diameter of 150 cm was divided into four equal quadrants, one of which is the target quadrant where the platform is placed, and the platform is located 1 cm below the water surface. The pool was surrounded by blue curtains and the water temperature was maintained at 21–22°C. Then, during the acquisition trial, the grouped mice were trained within 60 s for five consecutive days. The specific training steps were as follows: the numbered mice were randomly and quickly placed in different quadrants of the pool, and all four quadrants required training, and the time required for each mouse to find the hidden platform was recorded. Additionally, the behavior of the mice was observed through the monitoring system. If the mouse failed to find the platform within 60 s, the mouse would be guided to find the platform, and it would be taken away after familiarizing itself with the surrounding environment on the platform for 30 s, while the test time was recorded as 60 s. The escape latency of mice was recorded for each day during the training period. After 5 days of training, the hidden platform was removed on the last day, and a spatial probe test was performed. At this stage, mice were required to swim in the pool for 60 s, the number of platform crossings and the time spent in target quadrant were then recorded using ANY‐maze software (Stoelting).

### 2.5 Immunofluorescence

The details of immunofluorescence for brain sections have been described.^32^ Firstly, the mice were fully perfused with 4% PFA, and then, brains were rapidly stripped out after perfusion with cold normal saline. Subsequently, the brains were fixed by immersion in 4% formalin, then dehydrated and cryosectioned. The thickness of brain slices was set to 20 μm. After blocking in 2% BSA at room temperature for 2 h, brain sections were incubated overnight at 4°C with the following primary antibodies: anti‐CDK5 (ab40773, Abcam, rabbit, 1:200), anti‐PSD95 (ab18258, Abcam, rabbit, 1:200), and anti‐NeuN (ab104224, Abcam, mouse, 1:200). The next day, the primary antibody‐incubated brain slices were washed with PBS for 30 min and then incubated with fluorescent secondary antibodies diluted with 2% BSA at 1:500 for 2 h at room temperature. After several washes with PBS, cell nuclei were stained with DAPI reagent (1:1000, Bioworld) after several washes with PBS. At last, images were captured with a fluorescence microscope (Olympus IX73) or a confocal laser‐scanning microscope (Olympus FV3000). All qualitative immunostaining analyses were performed using ImageJ software (National Institutes of Health, USA).

### 2.6 TUNEL staining

Initially, on P14 and P70, brain tissues were collected from 3 to 4 mice in each group and fixed in 4% PFA overnight for paraffin sectioning with a thickness of 3 μm, followed by TUNEL staining according to the manufacturer's instructions (Roche). The tissue sections were treated with proteinase K at room temperature after being deparaffinized with xylene and hydrated with gradient ethanol. Subsequently, brain tissue sections were incubated with TUNEL reaction solution for 1 h at 37°C. After fixation with formaldehyde solution containing 3% hydrogen peroxide and incubation with streptavidin peroxidase at 37°C for 30 min. Then, cell nuclei were counterstained with hematoxylin. Finally, the brain sections were imaged using the Olympus microscope (BX51), and ImageJ software (National Institutes of Health, USA) was used for quantitative analysis. Three slices of each hippocampal tissue were randomly selected and photographed at 40× and 200× magnification, respectively. Apoptosis rate of hippocampal CA1 and DG regions is the ratio of the number of TUNEL‐positive cells to the total number of cells in this region.

### 2.7 Golgi staining

As described previously,^33^ the brains were quickly dissected out from mice under deep anesthesia at P14 and P70. Then, these brain tissues were used for Golgi staining to detect the structure of dendritic spines. A fast Golgi staining kit (FD Neurotechnologies) was used for this experiment. According to the manufacturer's instructions, briefly, the brain tissues of mice were immersed in the mixed solution of A and B, which was configured in advance, for 2 weeks at room temperature. During this period, brain tissues were asked to kept in dark place and then were soaked in solution C for 5 days. The brain tissues were cut into 100 μm slices by cryostat microtome (Leica), and then, the tissue was attached to the gelatin‐coated glass slide. After drying in dark at room temperature, the slides were washed twice with double distilled water, then washed with the mixture of solution D and E plus double distilled water for 10 min, and then washed twice with double distilled water. Then in 50%, 75%, and 95% alcohol gradient dehydration, and finally in anhydrous ethanol four times dehydration, every 4 min. After dehydration, transparent, and sealed, images were captured with a microscope (Olympus BX51) under a ×100 oil objective. In order to quantify dendritic spines, the sequence images were generated to construct three‐dimensional images of dendritic spines. Three brain slices were randomly selected from each mouse for quantitative analysis. ImageJ software was used to analyze dendritic spines. Spine densities were estimated by counting the number of the second‐order dendritic branches numbers of spines per 10 μm of dendrite length in CA1 and DG regions.

### 2.8 Western blot analysis

Western blot was performed as we did previously.^34^ Bilateral hippocampal tissues were isolated from mouse brains, and the total protein of hippocampal tissues was obtained by adding lysis buffer, which contained phenylmethanesulfonyl and phosphatase inhibitor. Samples were homogenized by ultrasonic three times on ice at 70 Hz for 5 s each, followed by centrifugation at 15,000 *g*, 4°C for 15 min. After fully dissolving in ice for 30 min, the BCA Protein Assay Kit was used to determine the protein concentration of the supernatants. Afterward, the supernatant was thoroughly mixed with 5× sample loading buffer and heated at 100°C for 10 min. Equal amounts of protein were then used for acrylamide denaturing gels (SDS‐PAGE). Proteins were separated by 12% SDS‐PAGE and transferred to polyvinylidene fluoride membranes (Millipore, IPVH00010), followed by blocking with 7.5% nonfat dried milk in phosphate‐buffered saline with Tween‐20 (PBST) (pH 7.5, 10 mM Tris–HCl, 150 mM NaCl, and 0.1% Tween 20) for 1 h at room temperature. After being washed several times with PBST buffer, the membranes were incubated overnight at 4°C with the following primary antibody: anti‐CDK5 (ab40773, Abcam, rabbit, 1:1000); anti‐BDNF (ab108319, Abcam, rabbit, 1:1000); anti‐Bcl‐2‐associated X (Bax) (sc‐7480, Santa Cruz, mouse, 1:1000); anti‐Bcl‐2 (sc‐7382, Santa Cruz, mouse, 1:500); anti‐cleaved caspase‐3 (#9664, Cell Signaling Technology, rabbit, 1:1000); anti‐Tubulin (BS1482M, Bioworld, mouse, 1:5000); anti‐Synaptophysin (SYP) (ab32127, Abcam, rabbit, 1:50,000); and anti‐PSD95 (ab18258, Abcam, rabbit, 1:1000). On the second day, the membranes were washed 30 min with PBST buffer and then further incubated for 2 h with horseradish peroxidase (HRP)‐labeled secondary antibody [Goat Anti‐Mouse IgG(H + L) HRP (1:5000, Multi‐Science) or Goat Anti‐Rabbit IgG(H + L) HRP (1:5000, Multi‐Science)] at room temperature. The membranes were then developed using enhanced chemiluminescence and subsequently were scanned with a gel imager (ChemiDOCTM MP Imaging System [Bio‐Rad]), and the densitometry of proteins was measured using Image LabTM software (Bio‐Rad). Relative expression of proteins is expressed as to be evaluated. The relative expression of the target protein was evaluated by the gray value ratio of the protein to Tubulin.

### 2.9 Statistical analysis

Statistics were performed using the GraphPad Prism 8.3.0 software, and all data were presented as mean ± SEM in the figure legend. Shapiro–Wilk test was used to test the normality assumption of the data. Student's *t*‐test was used to compare differences between two groups if the data were normal distribution, while Mann–Whitney test was applied to compare the non‐normally distributed variables. For more than two groups, statistical difference was analyzed by one‐way analysis of variance (ANOVA) followed by Bonferroni's post hoc test or by the Kruskal–Wallis test followed by Dunn's multiple comparison test. Meanwhile, two‐way ANOVA was used to analyze the significance of escape latency in the Morris water maze, followed by Bonferroni's post hoc test. *p* < 0.05 was considered to be statistically significant.

## 3 RESULTS

### 3.1 Long‐term learning and memory dysfunction in hyperoxia‐induced BPD mice

**FIGURE 1:**
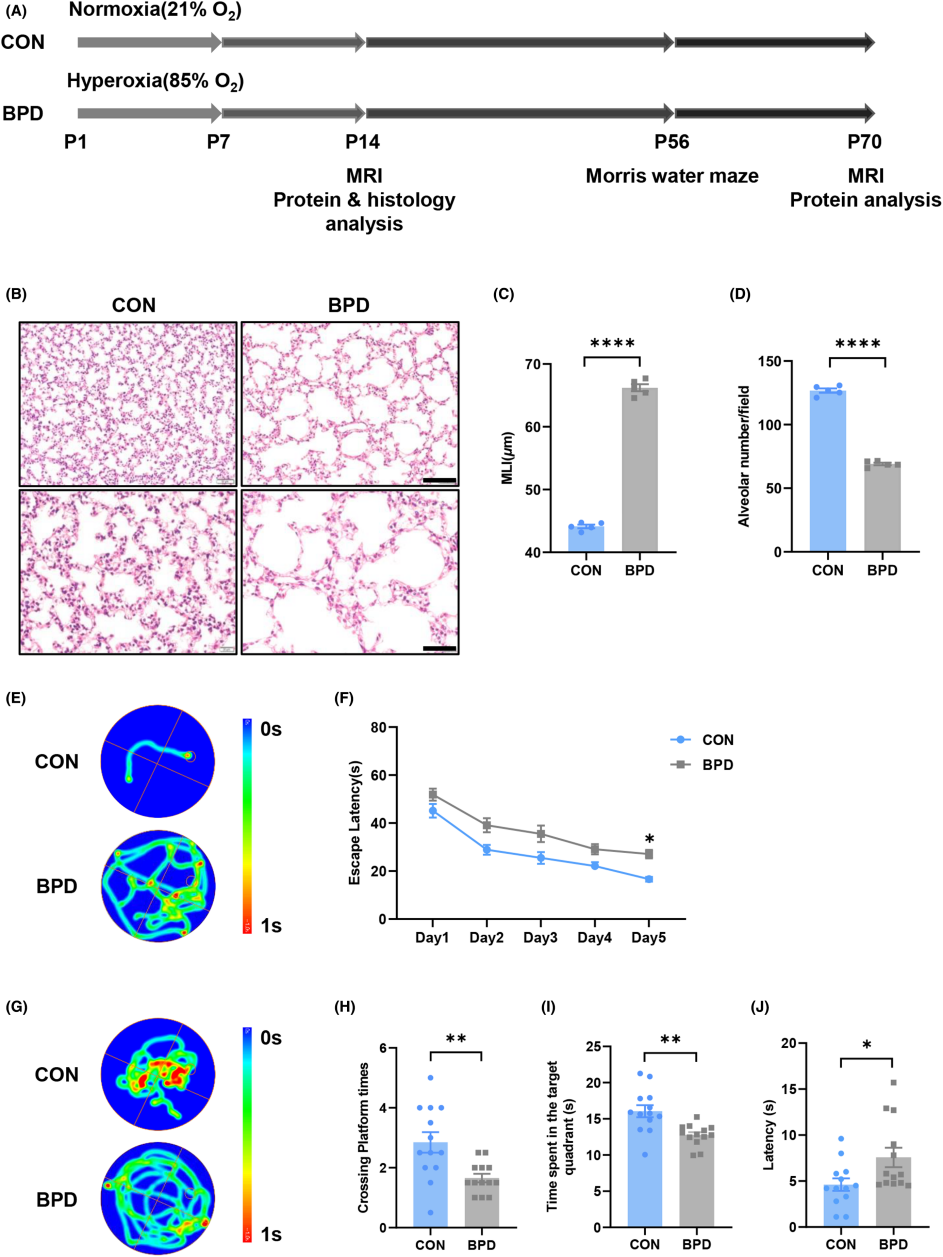
Long‐term learning and memory dysfunction in BPD mice. (A) Experimental design for B–J. (B) HE staining of lung tissue of P14 mice. Upper panel scale bar = 100 μm; lower panel scale bar = 50 μm. (C) The mean linear intercept of CON and BPD group. *n* = 5 for each group. (D) The alveolar number of CON and BPD group. *n* = 5 for each group. (E–J) Scatter plots showing representative motion curves of escape latency acquisition trial (E). The escape latency of the acquisition trial (F), representative motion curves of probe trial (G), crossing platform times (H), time spent in target quadrant (I), and latency to the target quadrant (J) in the probe trial in the MWM tests. *n* = 13 for each group. *p < 0.05, **p < 0.01, ****p < 0.0001.

In order to detect the effects of bronchopulmonary dysplasia on long‐term neurodevelopment in premature infants, a mouse model of BPD was constructed and Morris water maze test was performed to evaluate spatial learning and memory ability in adulthood (Figure 1A). Lung histomorphological analysis of mice on postnatal day 14 showed that the degree of alveolarization in mice exposed to hyperoxia was significantly reduced compared with controls (Figure 1B–D; *t* = 35.06, *p* < 0.0001 in Figure 1C; *t* = 29.18, *p* < 0.0001 in Figure 1D), which was consistent with the clinicopathological changes of BPD. The above results indicated that the BPD mouse model was successfully constructed in this experiment. Subsequently, the mice after modeling were reared to 8 weeks old for Morris water maze test. During the acquisition trial, the escape latency of the mice in the BPD group was significantly increased compared with the control group (Figure 1E,F; post hoc test following ANOVA, *p* = 0.0110 for CON vs. BPD at Day 5 in Figure 1F). In the spatial probe trial, as compared to the control group, BPD group mice had significantly fewer crossing platform times and spent less time in the target quadrant, and latency to target quadrant was remarkably increased (Figure 1G–J; *t* = 3.220, *p* = 0.0037 in Figure 1H; *t* = 3.559, *p* = 0.0016 in Figure 1I; *t* = 2.351, *p* = 0.0273 in Figure 1J). These results demonstrated that the BPD mouse model induced by hyperoxia had long‐term learning and memory dysfunction.

**FIGURE 2:**
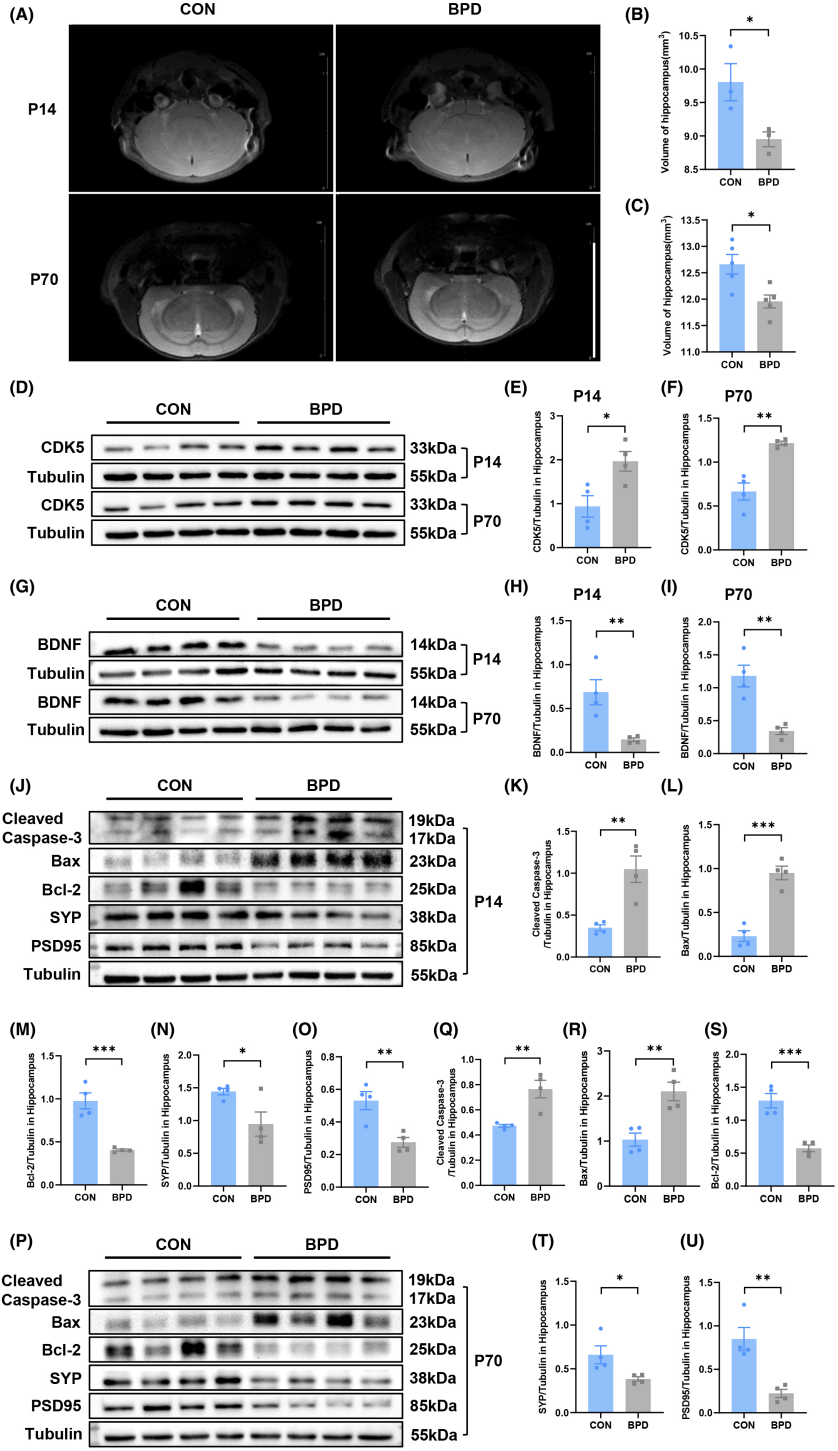
BPD induces decreased hippocampal volume and abnormal expression of CDK5 and BDNF and alters apoptosis‐related proteins and neuroplasticity‐related proteins expression. (A) Representative MRI images of hippocampus. Upper two images and lower two images are coronal sections at the level of the hippocampus of P14 and P70 mice respectively. Scale bar = 1 cm. (B) Volume of hippocampus of CON and BPD group at P14. *n* = 3 for each group. (C) Volume of hippocampus of CON and BPD group at P70. *n* = 5 for each group. (D–F) Representative blots (D) and scatter plot (E, F) showing CDK5 expression in hippocampus at P14 and P70. *n* = 4 for each group. (G–I) Representative blots (G) and scatter plot (H, I) showing BDNF expression in hippocampus at P14 and P70. *n* = 4 for each group. (J–O) Representative blots (J) and scatter plot (K–O) showing cleaved caspase‐3 (K), Bax (L), Bcl‐2 (M), SYP (N), PSD95 (O) expression in hippocampus at P14. (P–U) Representative blots (P) and scatter plot (Q–U) showing cleaved caspase‐3 (Q), Bax (R), Bcl‐2 (S), SYP (T), PSD95 (U) expression in hippocampus at P70. *n* = 4 for each group. *p < 0.05, **p < 0.01, ***p < 0.001.

### 3.2 Hippocampal volume in mice with BPD is reduced in both infancy and adulthood, while the expression of CDK5 and BDNF in hippocampus are abnormal

Subsequently, in order to evaluate whether BPD mice had structural abnormalities in the hippocampal region, brain MRI was performed (Figure 2A). The MRI results showed that the volume of the hippocampus of BPD mice was significantly reduced compared with that of the control group, both in the early brain development stage of the juvenile and in the brain mature stage of the adult (Figure 2B,C; *t* = 2.859, *p* = 0.0460 in Figure 2B; *t* = 3.153, *p* = 0.0135 in Figure 2C). In addition, our results demonstrated that the expression of CDK5 protein in the hippocampus of BPD mice was greatly increased compared with the control group at P14 and P70 (Figure 2D–F; *t* = 3.093, *p* = 0.0213 in Figure 2E; *t* = 5.503, *p* = 0.0015 in Figure 2F), while the expression of BDNF protein in the hippocampus of BPD mice was remarkably decreased (Figure 2G–I; *t* = 3.753, *p* = 0.0095 in Figure 2H; *t* = 4.850, *p* = 0.0029 in Figure 2I).

### 3.3 BPD alters the expression of apoptosis‐related proteins and neuroplasticity‐related proteins in hippocampus

Given the important role of CDK5 in regulating apoptosis and neuronal synaptic plasticity,^35^ we detected the expression levels of apoptosis and neuroplasticity‐related proteins in the hippocampus of BPD mice. When the BPD model was constructed on P14, the expression of apoptosis‐related proteins cleaved caspase‐3 and Bax in the hippocampus of the experimental group was notably higher than these of the control group, while the expression of anti‐apoptotic protein Bcl‐2 and neuroplasticity‐related proteins SYP and postsynaptic density protein 95 (PSD95) decreased significantly after successful disease induction (Figure 2J–O; *t* = 4.303, *p* = 0.0051 in Figure 2K; *t* = 7.337, *p* = 0.0003 in Figure 2L; *t* = 6.073, *p* = 0.0009 in Figure 2M; *t* = 2.585, *p* = 0.0415 in Figure 2N; *t* = 4.091, *p* = 0.0064 in Figure 2O). In order to clarify whether this effect had long‐term effects and was involved in the cognitive impairment of adult BPD mice, after the completion of the water maze tests in mice, we also detected the apoptosis and synaptic‐related proteins in the hippocampus of adult mice (P70). The results revealed that compared with the control group, the expression of cleaved caspase‐3 and Bax in the hippocampus of BPD mice was still significantly upregulated, while the expression of Bcl‐2, SYP, and PSD95 was strikingly downregulated (Figure 2P–U; *t* = 4.158, *p* = 0.0060 in Figure 2Q; *t* = 4.233, *p* = 0.0055 in Figure 2R; *t* = 5.995, *p* = 0.0010 in Figure 2S; *t* = 2.604, *p* = 0.0405 in Figure 2T; *t* = 4.466, *p* = 0.0043 in Figure 2U).

### 3.4 Inhibition of CDK5 ameliorates BPD‐associated cognitive deficits

**FIGURE 3:**
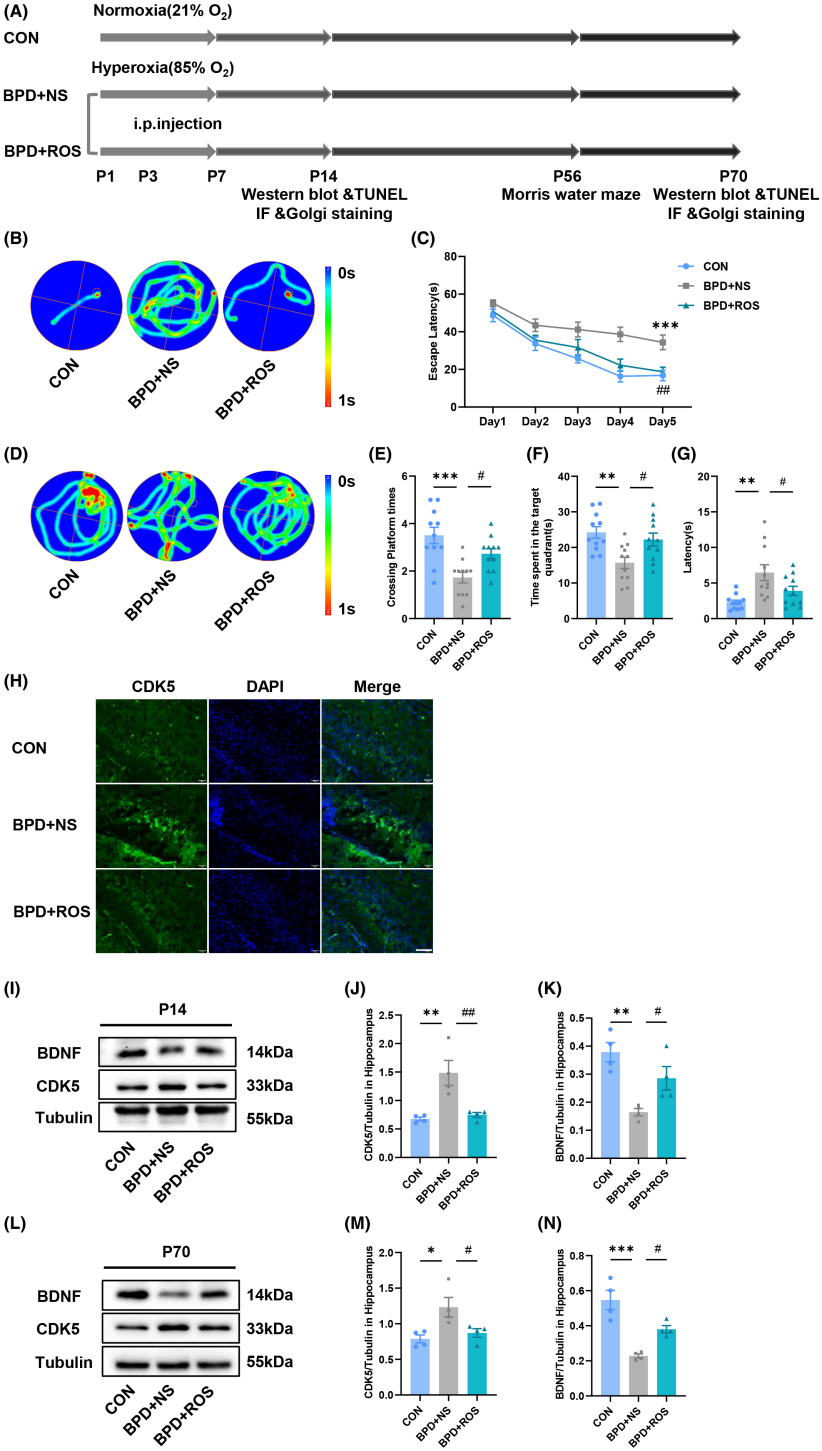
Inhibition of CDK5 alleviates BPD‐associated learning and memory impairment and reverses BPD‐associated BDNF downregulation. (A) Experimental design for (B–G). (B‐G) Scatter plots showing representative motion curves of escape latency of acquisition trial (B), the escape latency of the acquisition trial (C), representative motion curves of probe trial (D), crossing platform times (E), time spent in target quadrant (F), and latency to the target quadrant (G) in the probe trial in the MWM tests. *n* = 11 for each group. (H) Representative immunofluorescent images labeled with CDK5 in the DG area of hippocampus of CON, BPD + NS, and BPD + ROS mice at P14 were detected by immunofluorescence staining. Scale bar = 100 μm. (I–K) Representative blots (I) and scatter plot (J, K) showing CDK5 (J), BDNF (K) expression in hippocampus at P14. (L–N) Representative blots (L) and scatter plot (M, N) showing CDK5 (M), BDNF (N) expression in hippocampus at P70. *n* = 4 for each group. *p < 0.05, **p < 0.01, ***p < 0.001, ^#^ *p* < 0.05, ^##^ *p* < 0.01.

To investigate whether the upregulated CDK5 was involved in BPD‐related cognitive impairment, BPD mice were intraperitoneally injected on P3 and P7 with Roscovitine (ROS), a selective CDK5 inhibitor, which was able to play a role through the blood–brain barrier. The specific experimental process was shown in Figure 3A. Seven weeks after administration of the inhibitor, we evaluated the cognitive function of mice by MWM tests. Obviously, compared with the control group, the mice in the BPD + NS group spent more time searching for the underwater target platform during the acquisition trial, and this poor performance was remarkably improved in the BPD + ROS group (Figure 3B,C; *F*_(8,150)_ = 1.148, *p* = 0.3348 for interaction, *F*_(4,150)_ = 36.90, *p* < 0.0001 for treatment, *F*_(2,150)_ = 27.13, *p* < 0.0001 for days; *p* = 0.0003 for BPD + NS vs. CON and *p* = 0.0014 for BPD + NS vs. BPD + ROS at Day 5 in Figure 3C). During the spatial probe test, we observed that the crossing platform times and the time spent in the target quadrant in the BPD + NS group mice were obviously reduced compared with these in the control group, while the BPD + ROS group was significantly increased (Figure 3D–F; *F*_(2,30)_=10.92, *p* = 0.0003 for treatment; *p* = 0.0001 for BPD + NS vs. CON and *p* = 0.0249 for BPD + NS vs. BPD + ROS in Figure 3E; *F*_(2,30)_=7.071, *p* = 0.0030 for treatment; *p* = 0.0022 for BPD + NS vs. CON and *p* = 0.0186 for BPD + NS vs. BPD + ROS in Figure 3F). At the same time, for the latency to target quadrant, we found that the BPD + NS group was remarkably increased than that in the CON group, while that was decreased after ROS treatment (*F*_(2,30)_=7.535, *p* = 0.0022 for treatment; *p* = 0.0011 for BPD + NS vs. CON and *p* = 0.0454 for BPD + NS vs. BPD + ROS in Figure 3G).

### 3.5 Inhibition of CDK5 reverses BPD‐associated BDNF downregulation

BDNF, as a neurotrophic factor, is involved in the process of learning and memory.^36^ In order to further clarify whether CDK5 could affect the cognitive function of BPD mice by regulating the expression of BDNF, we first examined the expression of CDK5 in the hippocampus of BPD + NS mice by immunofluorescence and western blot after 1 week after the ROS treatment. The expression level of CDK5 was significantly upregulated in BPD + NS mice, compared with the control group, and ROS effectively inhibited the expression of CDK5 in the hippocampus of BPD mice (Figure 3H). Meanwhile, the protein expression levels of CDK5 and BDNF were detected 1 week after ROS treatment and in adulthood. The results showed that ROS treatment significantly reduced the expression of CDK5 in both P14 and P70 mice of BPD + NS group. In addition, BDNF expression in the hippocampus of BPD + NS group was significantly downregulated in both at P14 and adulthood. It was noteworthy that the application of ROS could effectively improve the expression of BDNF in both P14 and P70 mice with BPD (Figure 3I–N; *F*_(2,9)_ = 11.78, *p* = 0.0031 for treatment; *p* = 0.0033 for BPD + NS vs. CON and *p* = 0.0057 for BPD + NS vs. BPD + ROS in Figure 3J; *F*_(2,9)_ = 11.30, *p* = 0.0035 for treatment; *p* = 0.0020 for BPD + NS vs. CON and *p* = 0.0452 for BPD + NS vs. BPD + ROS in Figure 3K; *F*_(2,9)_ = 6.583, *p* = 0.0173 for treatment; *p* = 0.0141 for BPD + NS vs. CON and *p* = 0.0385 for BPD + NS vs. BPD + ROS in Figure 3M; *F*_(2,9)_ = 21.19, *p* = 0.0004 for treatment; *p* = 0.0002 for BPD + NS vs. CON and *p* = 0.0252 for BPD + NS vs. BPD + ROS in Figure 3N). The results demonstrated that the therapeutic effect of ROS had a long‐term effect.

### 3.6 Inhibition of CDK5 attenuates neuronal apoptosis in hippocampal CA1 and DG of BPD mice

**FIGURE 4:**
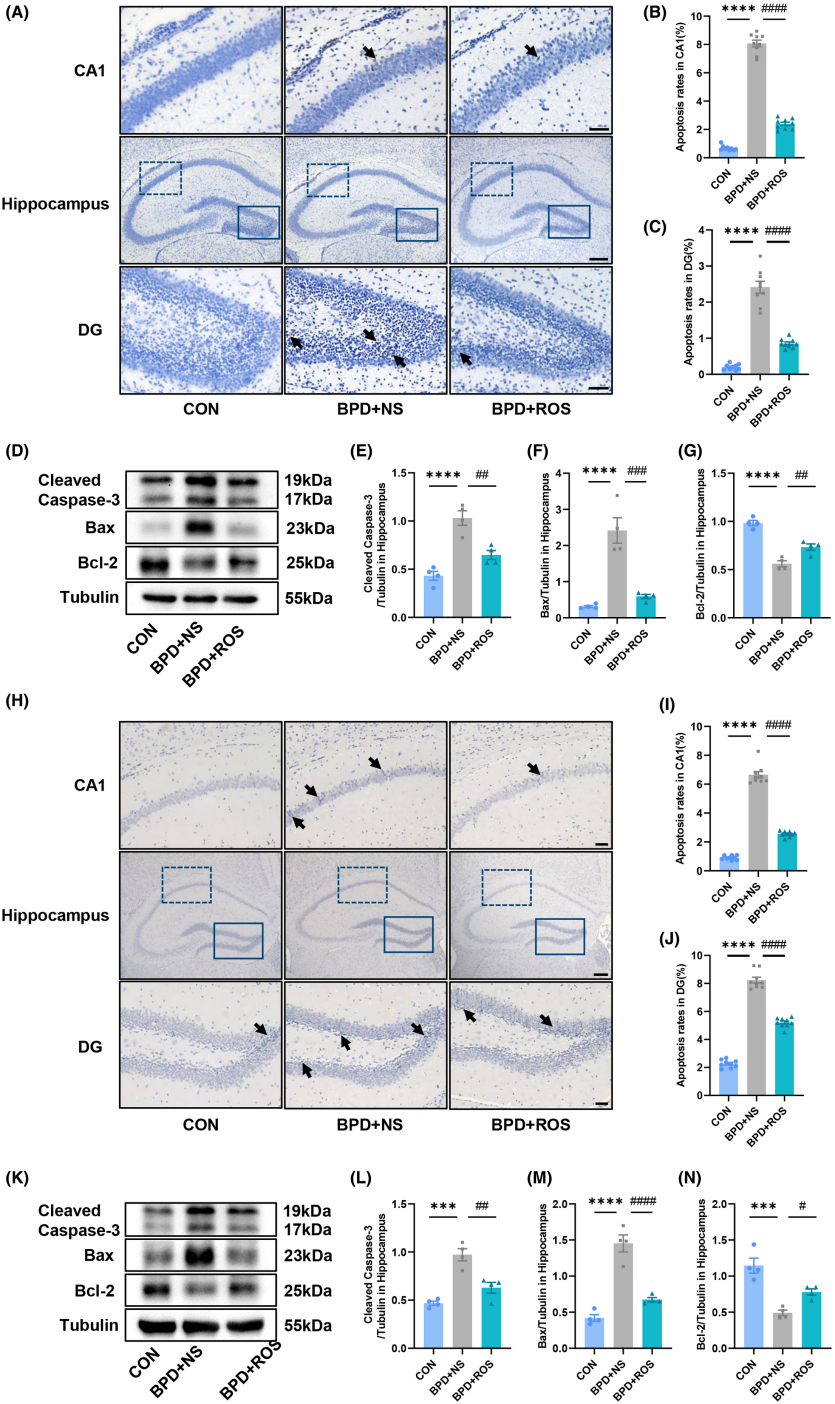
Inhibition of CDK5 ameliorates neuronal apoptosis in hippocampal CA1 and DG of BPD mice. (A) Representative images of TUNEL staining of hippocampal CA1 and DG of CON, BPD + NS, and BPD + ROS mice at P14. The arrows pointed to apoptotic cells. Upper and lower scale bar = 50 μm; Middle scale bar = 200 μm. (B, C) Apoptosis rates in hippocampal CA1 (B) and DG area (C) at P14. *n* = 9 slices from three mice in each group. (D–G) Representative blots (D) and scatter plot (E–G) showing cleaved caspaase‐3 (E), Bax (F), Bcl‐2 (G) expression in hippocampus at P14. *n* = 4 for each group. (H) Representative images of TUNEL staining of hippocampal CA1 and DG of CON, BPD + NS, and BPD + ROS mice at P70. The arrows pointed to apoptotic cells. Upper and lower scale bar = 50 μm; Middle scale bar = 200 μm. (I, J) Apoptosis rates in hippocampal CA1 (I) and DG area (J) at P70. *n* = 9 slices from three mice in each group. (K–N) Representative blots (K) and scatter plot (L‐N) showing cleaved caspase‐3 (L), Bax (M), Bcl‐2 (N) expression in hippocampus at P70. *n* = 4 for each group. ***p < 0.001, ****p < 0.0001, ^#^ *p* < 0.05, ^##^ *p* < 0.01, ^###^ *p* < 0.001, ^####^ *p* < 0.0001.

In previous experiments, we have observed that the expression of apoptosis‐related proteins cleaved caspase‐3 and Bax in the hippocampus of BPD mice was significantly increased compared with the control group, while the expression of anti‐apoptotic protein Bcl‐2 was significantly decreased. In order to further verify the close relationship between CDK5 and BPD‐related hippocampal injury, we not only detected the expression of cleaved caspase‐3, Bax, and Bcl‐2 by western blot after ROS treatment, but also observed the main apoptotic regions and measured the apoptosis ratio of the hippocampus of BPD mice by TUNEL staining. The results showed that at P14, the neuronal apoptosis ratio in the hippocampal CA1 and DG regions of the BPD + NS group was significantly higher than that of the control group, which could reduce by ROS. After ROS treatment, the TUNEL‐positive cell ratio in the CA1 region decreased from 8.070% to 2.391% in CA1, and from 2.413% to 0.854% in DG. (Figure 4A–C; *F*_(2,24)_ = 614.4, *p* < 0.0001 for treatment; *p* < 0.0001 for BPD + NS vs. CON and *p* < 0.0001 for BPD + NS vs. BPD + ROS in Figure 4B; *F*_(2,24)_ = 131.4, *p* < 0.0001 for treatment; *p* < 0.0001 for BPD + NS vs. CON and *p* = 0.0091 for BPD + NS vs. BPD + ROS in Figure 4C). At the same time, compared with the control group, the expression levels of cleaved caspase‐3 and Bax in the BPD + NS group were significantly increased, while Bcl‐2 was markedly decreased. ROS treatment could effectively improve these expression levels (Figure 4D–G; *F*_(2,9)_ = 27.54, *p* = 0.0001 for treatment; *p* < 0.0001 for BPD + NS vs. CON and *p* = 0.0021 for BPD + NS vs. BPD + ROS in Figure 4E; *F*_(2,9)_ = 30.90, *p* < 0.0001 for treatment; *p* < 0.0001 for BPD + NS vs. CON and *p* = 0.0003 for BPD + NS vs. BPD + ROS in Figure 4F; *F*_(2,9)_ = 47.18, *p* < 0.0001 for treatment; *p* < 0.0001 for BPD + NS vs. CON and *p* = 0.0062 for BPD + NS vs. BPD + ROS in Figure 4G). We also performed the same WB detection and TUNEL staining for P70 mice to evaluate the long‐term effect of ROS on apoptosis. Figure 4H–N showed that for BPD mice, after ROS treatment in P3 and P7, there was still significant therapeutic effect at P70. The data indicated that in adulthood, the ratio of TUNEL‐positive cells in hippocampal CA1 and DG of BPD mice increased significantly, from 0.905% to 6.650% in the CA1 and from 2.265% to 8.236% in the DG area. After ROS treatment, the apoptosis ratio decreased to 2.548% and 5.213%, respectively (*F*_(2,24)_ = 462.5, *p* < 0.0001 for treatment; *p* < 0.0001 for BPD + NS vs. CON and *p* < 0.0001 for BPD + NS vs. BPD + ROS in Figure 4I; *F*_(2,24)_ = 416.9, *p* < 0.0001 for treatment; *p* < 0.0001 for BPD + NS vs. CON and *p* < 0.0001 for BPD + NS vs. BPD + ROS in Figure 4J). Moreover, the expression trend of cleaved caspase‐3, Bax, and Bcl‐2 at P70 were consistent with those at P14 (*F*_(2,9)_=26.43, *p* = 0.0002 for treatment; *p* = 0.0001 for BPD + NS vs. CON and *p* = 0.0017 for BPD + NS vs. BPD + ROS in Figure 4L; *F*_(2,9)_=51.50, *p* < 0.0001 for treatment; *p* < 0.0001 for BPD + NS vs. CON and *p* < 0.0001 for BPD + NS vs. BPD + ROS in Figure 4M; *F*_(2,9)_=22.94, *p* = 0.0003 for treatment; *p* = 0.0002 for BPD + NS vs. CON and *p* = 0.0280 for BPD + NS vs. BPD + ROS in Figure 4N).

### 3.7 Inhibition of abnormal CDK5 improves neuronal synaptic plasticity in hippocampal CA1 and DG of BPD mice

**FIGURE 5:**
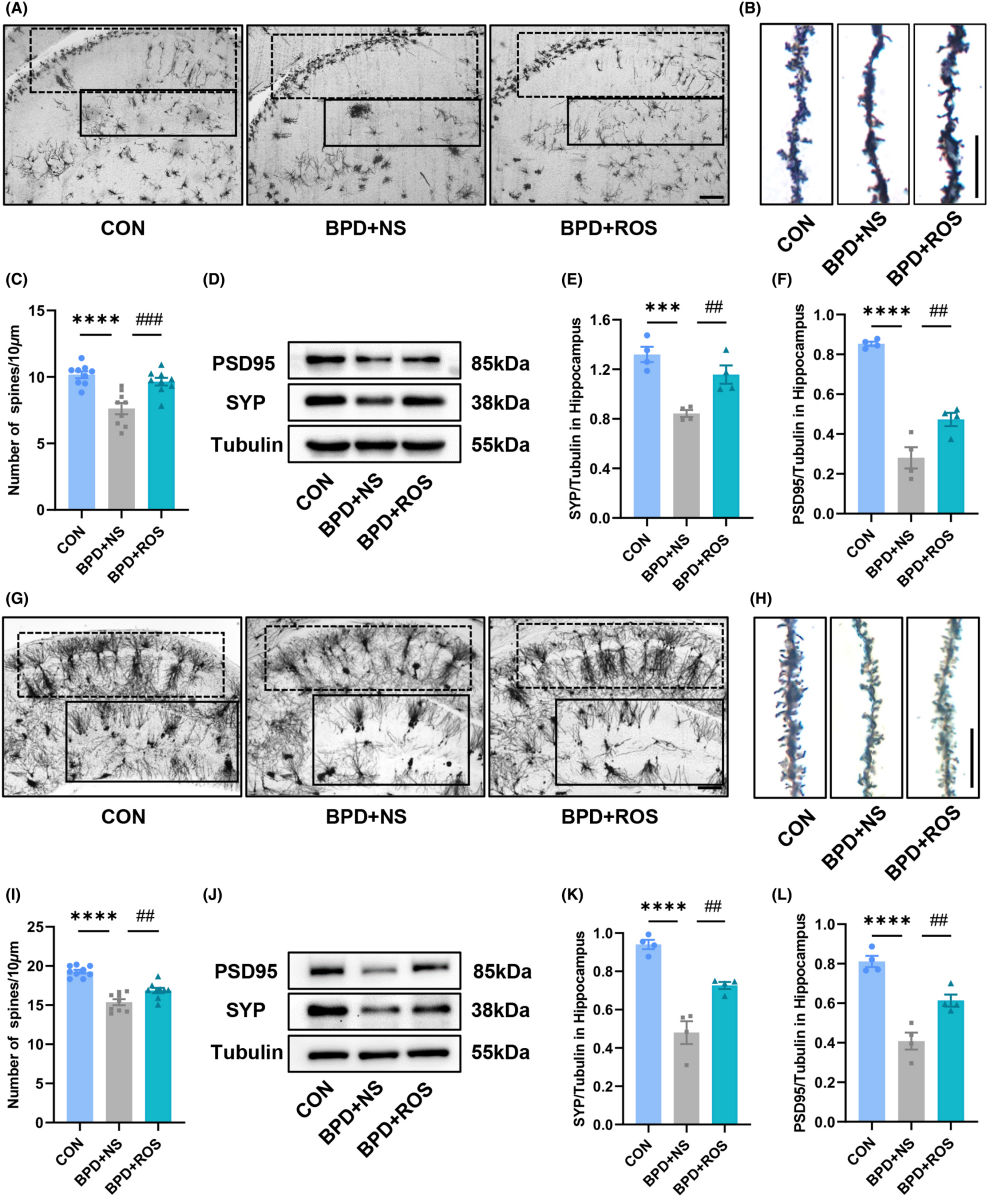
Inhibition of CDK5 improves synaptic plasticity in hippocampal CA1 and DG neurons of BPD mice. (A, B) Representative images of dendritic spines in the CA1 (dashed line) and DG (solid line) of hippocampus of three groups at P14 depicted in the Golgi staining. Scale bar = 100 μm (A), Scale bar = 10 μm (B). (C) Quantification of spine density. *n* = 9 slices from three mice in each group. (D–F) Representative blots (D) and scatter plot (E, F) showing SYP (E) and PSD95 (F) expression at P14. *n* = 4 for each group. (G, H) Representative images of dendritic spines in the CA1 (dashed line) and DG (solid line) of hippocampus of three groups at P70 depicted in the Golgi staining. Scale bar = 100 μm (G), Scale bar = 10 μm (H). (I) Quantification of spine density. *n* = 9 slices from three mice in each group. (J–L) Representative blots (J) and scatter plot (K, L) showing SYP (K) and PSD95 (L) expression at P70. *n* = 4 for each group. ***p < 0.001, ****p < 0.0001, ^##^ *p* < 0.01, ^###^ *p* < 0.001.

Neuronal synaptic plasticity is the biological basis of learning and memory formation.^37^ According to the previous experiments, the expression of synapse‐related proteins in the BPD group was significantly decreased. Therefore, we conducted a further study. Firstly, we performed Golgi staining on the brain slices of P14 mice. Compared with the control group, the developmental structure of neurons in the CA1 and DG areas of mice in the BPD + NS group was more disordered, and the number of neurons was reduced. However, the abnormal development of dendritic spines could be improved by ROS treatment. Through the quantitative analysis of dendritic spine density in the CA1 and DG areas, the data showed that the density of dendritic spines in the hippocampal CA1 and DG areas of the mice in the BPD + NS group was significantly lower than that in the control group. After ROS treatment, the density of dendritic spines was significantly improved (Figure 5A–C; *F*_(2,24)_ = 16.56, *p* < 0.0001 for treatment; *p* < 0.0001 for BPD + NS vs. CON and *p* = 0.0004 for BPD + NS vs. BPD + ROS in Figure 5C). In addition, we detected synapse‐related proteins. The expression levels of SYP and PSD95 in the hippocampus of mice in the BPD + NS group were significantly downregulated compared with those in the control group, while ROS treatment remarkably increased the expression levels of SYP and PSD95 (Figure 5D–F; *F*_(2,9)_ = 17.44, *p* = 0.0008 for treatment; *p* = 0.0005 for BPD + NS vs. CON and *p* = 0.0075 for BPD + NS vs. BPD + ROS in Figure 5E; *F*_(2,9)_ = 62.60, *p* < 0.0001 for treatment; *p* < 0.0001 for BPD + NS vs. CON and *p* = 0.0091 for BPD + NS vs. BPD + ROS in Figure 5F). Subsequently, the results of Golgi staining of brain sections further at P70 verified that ROS treatment could improve the synaptic plasticity of neurons in the hippocampal CA1 and DG areas of BPD mice. Under low magnification, we observed that the number of neuronal dendrites in the CA1 and DG areas of the adult mice in the BPD + NS group was lower than that of the CON group (Figure 5G), and then, the density of dendritic spines was statistically analyzed at high magnification (Figure 5H). The results demonstrated that the density of dendritic spines in the hippocampal CA1 and DG regions of mice in the BPD + NS group was still remarkably lower than that in the control group at adulthood, and this reduction could still be strikingly ameliorated after early ROS treatment (*F*_(2,24)_ = 38.08, *p* < 0.0001 for treatment; *p* < 0.0001 for BPD + NS vs. CON and *p* = 0.0058 for BPD + NS vs. BPD + ROS in Figure 5I). Meanwhile, at P70, ROS notably improved the expression levels of synapse‐related proteins in BPD mice consistent with that at P14 (*p* < 0.05; Figure 5J–L; *F*_(2,9)_=35.55, *p* < 0.0001 for treatment; *p* < 0.0001 for BPD + NS vs. CON and *p* = 0.0027 for BPD + NS vs. BPD + ROS in Figure 5K; *F*_(2,9)_=34.15, *p* < 0.0001 for treatment. Post hoc test following ANOVA, *p* < 0.0001 for BPD + NS vs. CON and *p* = 0.0042 for BPD + NS vs. BPD + ROS in Figure 5L).

### 3.8 Inhibition of CDK5 reverses BPD‐associated PSD95 downregulation in hippocampal DG and CA1

**FIGURE 6:**
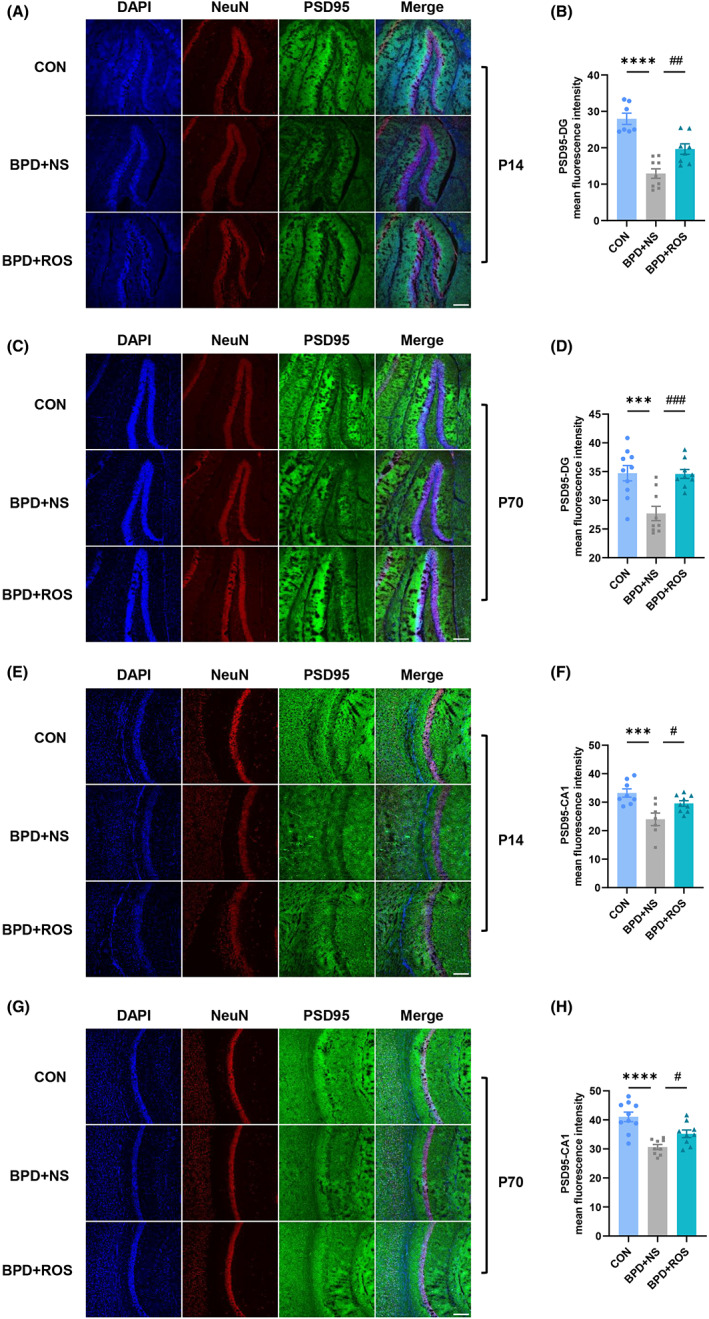
Inhibition of CDK5 reverses PSD95 downregulation in the hippocampal DG and CA1 regions of BPD mice. (A, B) Representative immunoblots and bar graph showing levels of PSD95 in the DG of hippocampus of CON, BPD + NS, and BPD + ROS mice at P14. *n* = 7–9 slices from three mice in each group. Scale bar = 200 μm. (C, D) Representative immunoblots and bar graph showing levels of PSD95 in the DG area of hippocampus of CON, BPD + NS, and BPD + ROS mice at P70. *n* = 9–10 slices from three mice in each group. Scale bar = 200 μm. (E, F) Representative immunoblots and bar graph showing levels of PSD95 in the CA1 of hippocampus of CON, BPD + NS, and BPD + ROS mice at P14. *n* = 7–9 slices from three mice in each group. Scale bar = 200 μm. (G, H) Representative immunoblots and bar graph showing levels of PSD95 in the CA1 area of hippocampus of CON, BPD + NS, and BPD + ROS mice at P70. *n* = 9–10 slices from three mice in each group. Scale bar = 200 μm. ***p < 0.001, ****p < 0.0001, ^#^ *p* < 0.05, ^##^ *p* < 0.01, ^###^ *p* < 0.001.

PSD95, an essential scaffolding protein, plays an important role in synaptic plasticity and dendritic spine morphogenesis.^38^ The immunofluorescence results showed that at P14, the mean fluorescence intensity of PSD95 of BPD + NS group in the DG area was significantly decreased compared with that in the CON group and ROS treatment apparently improved it (Figure 6A,B; *F*_(2,21)_ = 27.63, *p* < 0.0001 for treatment; *p* < 0.0001 for BPD + NS vs. CON and *p* = 0.0047 for BPD + NS vs. BPD + ROS in Figure 6B). It was worth noting that the mean fluorescence intensity of PSD95 of BPD + NS group in the DG area at P70 was inferior than the CON group and ROS treatment could enhance it (Figure 6C,D; *F*_(2,25)_ = 11.64, *p* = 0.0003 for treatment; *p* = 0.0005 for BPD + NS vs. CON and *p* = 0.0008 for BPD + NS vs. BPD + ROS in Figure 6D). Meanwhile, ROS treatment also improved the expression of PSD95 in the CA1 region of BPD + NS mice at P14 (Figure 6E,F; *F*_(2,21)_ = 8.561, *p* = 0.0019 for treatment; *p* = 0.0009 for BPD + NS vs. CON and *p* = 0.0337 for BPD + NS vs. BPD + ROS in Figure 6F) and P70 (Figure 6G,H; *F*_(2,25)_ = 15.39, *p* < 0.0001 for treatment; *p* < 0.0001 for BPD + NS vs. CON and *p* = 0.0480 for BPD + NS vs. BPD + ROS in Figure 6H).

## 4 DISCUSSION

In this study, we demonstrate that CDK5 in hippocampus is critical in BPD‐related brain injury. The increased CDK5 expression in BPD mice which conducted the expression of apoptosis‐related protein and synaptic plasticity‐related protein. More importantly, we explain the mechanism of CDK5 in BPD‐associated brain injury from the short‐term and long‐term effects, which is likely to be achieved by regulating BDNF. Inhibition of CDK5 expression by ROS can alleviate BPD‐associated hippocampal neuronal apoptosis and abnormal learning and memory. These data provide the first evidence that hyperoxia‐induced aberrant CDK5 activation is involved in the cognitive deficits associated with BPD brain injury.

Bronchopulmonary dysplasia (BPD), a chronic lung disease, caused by prolonged mechanical ventilation and hyperoxia in preterm infants.^39^ Clinical data show preterm infants with BPD are more likely to receive special education services than non‐BPD.^40^ In a clinical study with prospective follow‐up up to 8 years of age, the BPD group were found to have deficits in intelligence, reading, mathematics, and gross motor skills compared with the lower‐weight and full‐term infant groups.^41^ Thompson et al.^42^ discovered that BPD was associated with an overall reduction in gray and white matter regions. In multivariate analysis, BPD remained a significant predictor of delayed brain maturation,^43^ but it is unclear to what extent lung injuries contribute to brain injury in preterm infants, and there is no effective treatment for either condition.^44^ Many studies have confirmed that the hippocampus is an important brain region regulating learning and memory.^45^ In particular, during brain development, the brain is highly plastic. However, there are few studies on BPD brain injury in preterm infants and the underlining mechanisms connecting lung injury to brain injury are poorly understood. In our experiment, 85% hyperoxia which known to cause lung structural anomalies in premature mice pups established a mouse model of BPD in preterm infants. We found that the incubation period of mice in adult Morris water maze was significantly prolonged, and the mice showed significant learning and memory impairment (Figure 1E‐J).

CDK5 is a serine/threonine kinase with high activity in the brain.^46^ Unlike other CDK family members, it is not involved in the regulation of cell cycle, and its main function is the basis of neurotransmission and synaptic plasticity because its activator p35/p39 is expressed in neurons.^47^, ^48^ Abnormal CDK5 function is involved in the pathological process of many neurodegenerative diseases, including Alzheimer's disease,^49^ Parkinson's disease,^50^ and stroke.^51^ However, to our knowledge, no papers have been published about CDK5 associated with BPD brain injury. In the present study, we uniquely identified independent mechanism by which CDK5 regulates learning and memory in BPD in hippocampus, namely increased protein expression levels, and increased specific activity. Structural and functional plasticity is fundamental for mediating of learning and memory.^52^ For the first time, we evaluated the effects of CDK5 on synaptic plasticity in the hippocampal DG and neuronal apoptosis in the hippocampal CA1 of BPD mice in the short and long term. BDNF stands out for its high expression in the brain to have potent influence on synapses among all neurotrophins.^53^ Meanwhile, inhibition of CDK5 was found to significantly increase the expression of hippocampal BDNF to alleviate learning and memory impairments (Figure 3B‐N).

Our study still has some limitations. One of the limitations is that we did not record changes in electrophysiological activity of neurons in the hippocampal region of BPD mice, such as action potential, excitatory postsynaptic currents, and inhibitory postsynaptic currents. Thus, future studies are needed to determine whether specific neuron in a specific region of the hippocampus in hyperoxia‐injured neonatal mice. Another limitation of this study is that it did not conduct a detailed study of the hippocampal regions, such as the ventral and dorsal hippocampus. As we all know, the dorsal hippocampus performs cognitive functions,^54^ while ventral hippocampus is responsible for emotion and stress.^54^, ^55^ Therefore, according to the mature experimental skills of our laboratory,^34^ we next use the Cre‐Loxp system to artificially knock down the expression of CDK5 by injecting AAV‐hSyn‐Cre virus into the different hippocampal regions of CDK5^flox/flox^ mice by stereolocalization of brain regions to evaluate whether the pathological changes of BPD could be induced. Alternatively, the related mechanism of CDK5 downstream signaling pathway in BPD‐associated brain injury still needs to be explored.

Taken together, we demonstrated that hyperoxia exposure not only impaired lung, but also damaged the hippocampus of brain. These findings suggest that hippocampal CDK5 might be a more effective target for the treatment of cognitive deficits related with BPD. Although we proposed a new mechanism between lung injury and neurodevelopmental disorders in infants with BPD, more work is needed to elucidate the role of CDK5 in different brain regions in the regulation of BPD brain injury and to uncover the mechanisms involved.

## 5 CONCLUSION

This study is the first to reveal that CDK5 mediates BPD‐associated neurodevelopmental disorders in premature infants. Our study suggests that CDK5 is likely to cause abnormalities in the structure and functional development of dendritic spines by regulating neuroplasticity‐related proteins. Meanwhile, CDK5 promotes apoptosis of hippocampal neurons via modulating apoptosis‐related proteins, thereby downregulating BDNF is secondary to brain damage caused by bronchopulmonary dysplasia. Inhibition of CDK5 overexpression could effectively improve BPD‐associated abnormal neurodevelopment. Our findings support that hippocampal CDK5 may be a new target for the treatment of learning and memory dysfunction related to BPD.

## AUTHOR CONTRIBUTIONS

C.‐Y. Yin and R. Cheng designed research. F.‐F. Tao, Z.‐Y. Wang, Y. Wang, Q.‐R. Lv, P.‐P. Cai, H.‐W. Min, and J.‐W. Ge performed experiments. F.‐F. Tao analyzed the data and wrote the manuscript. C.‐Y. Yin and R. Cheng provided valuable comments and revised the manuscript. All authors read and approved the final version of the manuscript.

## CONFLICT OF INTEREST STATEMENT

The authors declare no competing conflicts of interest.

## Data Availability

The data supporting the findings of this study will be made available from the corresponding author upon reasonable request.
